# Effect of *BDNF* Val66Met on Memory Decline and Hippocampal Atrophy in Prodromal Alzheimer’s Disease: A Preliminary Study

**DOI:** 10.1371/journal.pone.0086498

**Published:** 2014-01-27

**Authors:** Yen Ying Lim, Victor L. Villemagne, Simon M. Laws, David Ames, Robert H. Pietrzak, Kathryn A. Ellis, Karra Harrington, Pierrick Bourgeat, Ashley I. Bush, Ralph N. Martins, Colin L. Masters, Christopher C. Rowe, Paul Maruff, for the AIBL Research Group

**Affiliations:** 1 Florey Institute of Neuroscience and Mental Health, The University of Melbourne, Parkville, Victoria, Australia; 2 Department of Nuclear Medicine and Centre for PET, Austin Health, Heidelberg, Victoria, Australia; 3 Department of Medicine, Austin Health, The University of Melbourne, Heidelberg, Victoria, Australia; 4 Centre of Excellence for Alzheimer’s Disease Research and Care, School of Medical Sciences, Edith Cowan University, Joondalup, Western Australia, Australia; 5 Sir James McCusker Alzheimer’s Disease Research Unit, Hollywood Private Hospital, Perth, Western Australia, Australia; 6 Co-operative Research Centre for Mental Health, Carlton South, Australia; 7 Academic Unit for Psychiatry of Old Age, Department of Psychiatry, The University of Melbourne, Kew, Victoria, Australia; 8 National Ageing Research Institute, Parkville, Victoria, Australia; 9 Department of Psychiatry, Yale University School of Medicine, New Haven, Connecticut, United States of America; 10 Commonwealth Scientific Industrial Research Organization Preventative Health National Research Flagship, Australian e-Health Research Centre-BioMedIA, Brisbane, Queensland, Australia; 11 CogState Ltd., Melbourne, Victoria, Australia; Institution of Automation, CAS, China

## Abstract

**Objective:**

Cross-sectional genetic association studies have reported equivocal results on the relationship between the brain-derived neurotrophic factor (*BDNF*) Val66Met and risk of Alzheimer’s disease (AD). As AD is a neurodegenerative disease, genetic influences may become clearer from prospective study. We aimed to determine whether *BDNF* Val66Met polymorphism influences changes in memory performance, hippocampal volume, and Aβ accumulation in adults with amnestic mild cognitive impairment (aMCI) and high Aβ.

**Methods:**

Thirty-four adults with aMCI were recruited from the Australian, Imaging, Biomarkers and Lifestyle (AIBL) Study. Participants underwent PiB-PET and structural MRI neuroimaging, neuropsychological assessments and BDNF genotyping at baseline, 18 month, and 36 month assessments.

**Results:**

In individuals with aMCI and high Aβ, Met carriers showed significant and large decline in episodic memory (*d* = 0.90, *p* = .020) and hippocampal volume (*d* = 0.98, *p* = .035). *BDNF* Val66Met was unrelated to the rate of Aβ accumulation (*d* = −0.35, *p* = .401).

**Conclusions:**

Although preliminary due to the small sample size, results of this study suggest that high Aβ levels and Met carriage may be useful prognostic markers of accelerated decline in episodic memory, and reductions in hippocampal volume in individuals in the prodromal or MCI stage of AD.

## Introduction

Current models of Alzheimer’s disease (AD) emphasise beta-amyloid (Aβ) as precipitating a cascade of events that result in synaptic loss and memory impairment [1]. Recent *in vitro* evidence suggests neurotrophic factors such as brain-derived neurotrophic factor (BDNF) may be an indirect moderator of Aβ neurotoxicity, as BDNF and its main receptor, tropomyosin-related kinase B (TrkB) are not involved in amyloidogenesis, but rather in synaptic excitation and neuronal plasticity, which may provide an ability for the central nervous system (CNS) to withstand Aβ-related neuronal death [2−4]. Further, in individuals with AD or mild cognitive impairment (MCI), BDNF messenger ribonucleic acid (mRNA) is reduced substantially in the hippocampus and temporal lobe, with the extent of BDNF loss associated with the magnitude of cognitive impairment [5], [6]. Unfortunately, there are currently no validated peripheral markers of central nervous system (CNS) BDNF [3], [4]. Therefore, in humans, conclusions about the role of BDNF in AD have been based on the measurement of the effect of *BDNF* polymorphisms (e.g., Val66Met [rs6265]) on clinical or pathological features of the disease, or on risk for AD [3], [4]. Unfortunately, evidence from such studies has been mixed with some showing *BDNF* ^Met^ carriers to have increased memory impairment, brain atrophy or risk of AD while others show that these same impairments are associated with *BDNF* ^Val^ homozygosity [3], [4], [7].

We [8] and others [3] have argued that the inconsistency of relationships between the *BNDF* Val66Met polymorphism and AD in human studies suggest the involvement of a moderating factor. As a proportion of healthy individuals at risk for AD have high Aβ [9], [10], and healthy individuals with high Aβ show substantial decline in episodic memory as well as increased hippocampal atrophy [10−12]_ENREF_5, the effects of *BDNF* polymorphisms may be moderated by Aβ levels. Recently, we observed an epistatic relationship between the *BDNF* Val66Met polymorphism and Aβ deposition in which *BDNF* ^Met^ healthy individuals showed a faster rate of hippocampal atrophy and episodic memory decline than *BDNF* ^Val^ homozygotes, but only if they had abnormally high Aβ [8]. Thus, this suggests that Aβ deposition may moderate the relationship between the *BDNF* Val66Met polymorphism and risk of AD.

Objective but subtle memory impairment with corroborating evidence of memory difficulties from a reliable informant is codified as the clinical classification mild cognitive impairment (MCI), and is associated with increased risk of progression to AD [12−14]. This risk is increased if the MCI classification is accompanied by the presence of abnormal Aβ levels [14]. Therefore, another test of the hypothesis that the *BDNF* Val66Met polymorphism increase risk of AD would be if individuals with MCI and high Aβ who are *BDNF* ^Met^ carriers show greater decline in episodic memory and hippocampal volume than *BDNF* ^Val^ homozygotes. However, as BDNF is an indirect mediator of Aβ toxicity, a second hypothesis is that the *BDNF* Val66Met polymorphism will not affect Aβ accumulation.

## Methods

### Participants

Thirty-four adults with aMCI and high Aβ levels enrolled in the Australian Imaging, Biomarkers and Lifestyle (AIBL) Study were included in this study [9], [15]. Participants had undergone *BDNF* genotyping at baseline, and positron emission tomography (PET) neuroimaging using Pittsburgh Compound B (PiB), structural magnetic resonance imaging (MRI), and neuropsychological assessment at baseline, 18 and 36 month follow-up (Table 1). The process of recruitment and diagnostic classification of adults with aMCI enrolled in the AIBL cohort has been described in detail previously [15]. Participants who volunteered were excluded from the AIBL study if they had any of the following: schizophrenia; depression (Geriatric Depression Score (GDS) of 6 or greater); Parkinson’s disease; cancer (other than basal cell skin carcinoma) within the last two years; symptomatic stroke; uncontrolled diabetes; or current regular alcohol use exceeding two standard drinks per day for women or four per day for men. A clinical review panel chaired by DA reviewed all available medical, psychiatric and neuropsychological information to ensure that their clinical classification was consistent with international criteria [16], [17]. Clinical classification was blinded to Aβ imaging data. The AIBL study was approved by the institutional ethics committees of Austin Health, St Vincent's Health, Hollywood Private Hospital and Edith Cowan University [15]. All participants with MCI and their caregivers provided written informed consent prior to being tested.

**Table 1:** Demographic means (SD) for MMSE, CDR-SB, premorbid IQ and HADS scores, and median years of education, for overall and each BDNF group at baseline assessment.

	Overall (n = 34)	*BDNF* ^Val^ homozygote (n = 24)	*BDNF* ^Met^ carrier (n = 10)	*p*
N (%) female	17 (50%)	12 (50%)	5 (50%)	1.00
N (%) *APOE* ε4	28 (82%)	19 (79%)	9 (90%)	.450
Age (years)	76.41 (6.36)	75.33 (5.47)	79.00 (7.83)	.127
SUVR Neocortex	2.22 (0.41)	2.14 (0.36)	2.39 (0.49)	.113
MMSE	27.03 (1.90)	27.08 (1.77)	26.90 (2.28)	.802
CDR-SB	1.03 (0.75)	1.04 (0.82)	1.00 (0.56)	.890
Premorbid IQ	109.09 (7.03)	107.63 (7.32)	112.60 (4.97)	.059
HADS-Depression	3.50 (2.44)	3.54 (2.60)	3.40 (2.12)	.880
HADS-Anxiety	4.82 (2.52)	4.71 (2.56)	5.10 (2.51)	.113

Note: One-Way ANOVA indicated no significant differences between Val/Val homozygotes and Met carriers on any demographic or clinical characteristic, *p*’s <.05. χ^2^ indicated that number of *APOE* ε4 carriers, *p*  = .45, and number of females, *p* = 1.00, were not higher in Met carriers.

Standardized Uptake Value Ratio; MMSE  =  Mini-Mental State Examination; CDR-SB  =  Clinical Dementia Rating Scale, Sum of Boxes Score; HADS-Depression  =  Hospital Anxiety and Depression Scale, Depression Subscale; HADS-Anxiety  =  Hospital Anxiety and Depression Scale, Anxiety Subscale.

### Measures

**Neuroimaging.** PiB-PET imaging was conducted as described previously [9], [10]. PET standardized uptake value (SUV) data acquired 40-70 minutes post-PiB injection were summed and normalized to the cerebellar cortex SUV, resulting in a region-to-cerebellar ratio termed SUV ratio (SUVR).

Magnetic resonance (MR) images were spatially normalized to the Montreal Neurological Institute (MNI) single-subject MRI brain template, [18] using MilxView®, software developed by the Australian e-Health Research Centre – BioMedIA (Brisbane, Australia). As described elsewhere, T1W MR images for each subject were classified into grey matter (GM), white matter (WM) and CSF using an implementation of the expectation maximization segmentation algorithm[19]. The algorithm computed probability maps for each tissue type and was used to assign each voxel to its most likely tissue type and subsequent segmentation. To improve the accuracy of analysis of the hippocampus, a separate, manually-delineated template was drawn on the MNI single-subject every 1 mm on coronal slices, and was subsequently used for hippocampal volume. The average hippocampal volumes were normalized for head size using the total intracranial volume, defined as the sum of GM, WM and CSF volumes.

**Genotyping.** An 80 ml blood sample was taken from each participant and 10 ml was used for large scale DNA extraction for AIBL bio-banking. The *BDNF* Val66Met polymorphism (rs6265) was included in a custom Illumina GoldenGate assay, which included 1536 SNPs, and was performed by the Beijing Genomics Institute. Val66Met polymorphism had a call rate of greater than 99% and did not depart from Hardy-Weinberg equilibrium in the AIBL aMCI group.

**Clinical and cognitive assessments.** The AIBL clinical and cognitive battery has been described in detail elsewhere and were administered according to standard protocols by trained research assistants [15], although the current study focused only on data for episodic memory. Clinical status was determined using information which included the Mini-Mental Status Examination (MMSE) and Clinical Dementia Rating (CDR) Scale. Premorbid intelligence was estimated using the Wechsler Test of Adult Reading (WTAR), and depressive and anxiety symptoms were assessed using the Hospital Anxiety and Depression Scale (HADS).

### Data Analysis

The episodic memory composite was computed by standardizing outcome measures on individual tests (Logical Memory delayed recall, California Verbal Learning Test, Second Edition [CVLT-II] delayed recall) against the baseline mean and SD for the entire group and then averaging them. As in previous studies [9], [10], adults with aMCI were classified as having high Aβ if they had an SUVR≥1.5. Linear mixed model (LMM) analyses of covariance were conducted to compare linear slopes of change in episodic memory, hippocampal volume and SUVR between *BDNF* groups across baseline, 18 and 36 month assessments. Linear mixed modelling was employed because of its ability to model both fixed and random effects, which accounts for multiple sources of variability, and because it provides improved estimates of within-subject coefficients (i.e., random effects) in longitudinal studies. For each LMM, *BDNF*, time, and *BDNF*-time interaction were entered as fixed factors; participant as a random factor; age and *APOE* status as covariates; and episodic memory, hippocampal volume or Aβ accumulation as dependent variables. For each outcome, the magnitude of the difference in slopes between the *BDNF* Val66Met polymorphism groups was expressed using Cohen’s *d*.

## Results

Demographic and clinical characteristics of the total sample and *BDNF* Val66Met polymorphism groups are shown in Table 1. Study groups were matched on all demographic and clinical characteristics.

**Figure 1:**
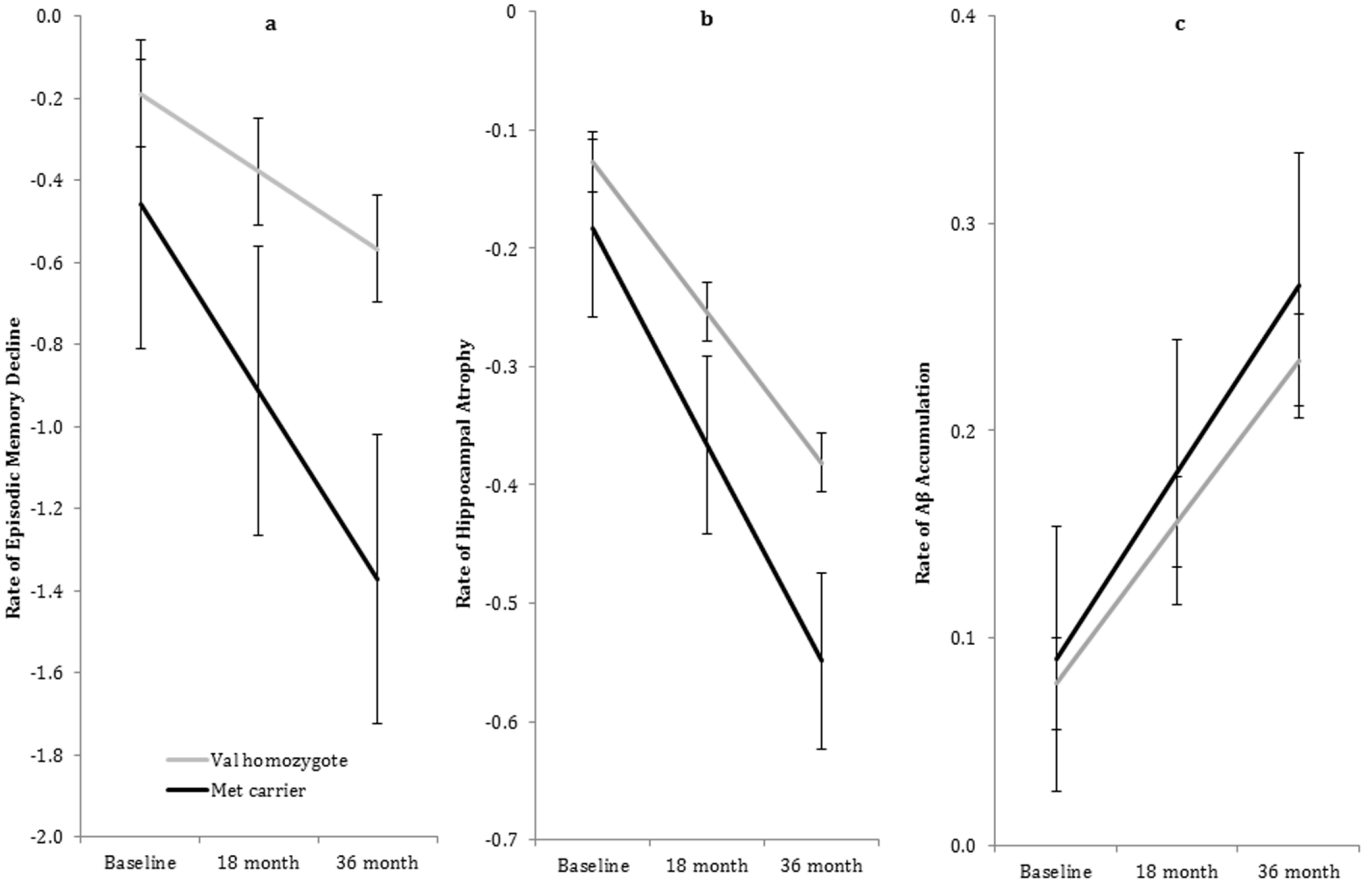
Trajectories of change in Episodic Memory Composite (1a), Hippocampal volume atrophy (1b), and Aβ accumulation (1c) for Val homozygotes and Met carriers (error bars represent 95% confidence intervals).

Relative to *BDNF* ^Val^ homozygotes, *BDNF* ^Met^ carriers showed a greater rate of decline in episodic memory and reduction in hippocampal volume over 36 months (Table 2, Figure 1a, 1b), with the effect size of difference between slopes for both measures, large in magnitude. Groups did not differ in the rate of Aβ accumulation over 36 months (Table 2, Figure 1c).

**Table 2:** Mean slopes (SD) for each neuropsychological measure, and magnitude of difference (Cohen’s d) in slopes.

	Episodic Memory		Hippocampal Atrophy		Aβ accumulation	
	(df) F	*p*	(df) F	*P*	(df) F	*p*
Age	(1,34) 0.312	.580	(1,32) 1.042	.315	(1,35) 4.538	.040
*APOE*	(1,33) 1.242	.273	(1,31) 2.951	.096	(1,33) 1.437	.239
Time	(1,24) 36.07	.000	(1,17) 166.041	.000	(1,20) 82.358	.000
*BDNF*	(1,28) 0.756	.392	(1,32) 5.412	.026	(1,34) 0.997	.325
*BDNF* x Time	(1,24) 6.228	.020	(1,17) 5.241	.035	(1,20) 0.735	.401
	Mean slope (SD)		Mean slope (SD)		Mean slope (SD)	
Aβ+ *BDNF* ^Val^ homozygote (n = 24)	−0.189 (0.310)		−0.128 (0.057)		0.079 (0.049)	
Aβ+ *BDNF* ^Met^ carrier (n = 10)	−0.457 (0.270)		−0.184 (0.057)		0.096 (0.046)	
Cohen’s *d* (95%CI)	0.90 (0.11, 1.64)		0.98 (0.19, 1.73)		−0.35 (−1.09, 0.40)	

## Discussion

The first hypothesis that the *BDNF* Val66Met polymorphism would moderate memory decline and hippocampal atrophy in aMCI with high Aβ was supported. In adults with aMCI for whom PiB-PET neuroimaging indicated high baseline Aβ, *BDNF* ^Met^ carriers showed greater episodic memory decline and hippocampal atrophy over 36 months compared to *BDNF* ^Val^ homozygotes, and the rate of decline between groups was, by convention, large in magnitude. While increased memory decline and hippocampal atrophy have been reported previously in adults with aMCI and high Aβ [10−12], results of the current study suggests a direct link between Aβ and the *BDNF* Val66Met polymorphism on progressive memory decline and hippocampal atrophy in prodromal AD.

The second hypothesis that the *BDNF* Val66Met polymorphism would not moderate the rate of Aβ accumulation in adults with aMCI and high Aβ was also supported. While Aβ levels increased for both *BDNF* ^Met^ carriers and *BDNF* ^Val^ homozygotes over 36 months, the rate of Aβ accumulation across 36 months was not different between groups (Figure 1c). Thus, while high baseline Aβ was associated with memory decline and hippocampal atrophy, the deleterious effects were reduced in individuals who carried the *BDNF* Val66Met polymorphism that has been associated with greater secretion of the BDNF protein (i.e., *BDNF* ^Val^ homozygotes).

The results of this study are consistent with our previous finding in healthy individuals, where *BDNF* ^Met^ carriers with high Aβ showed significantly higher rates of episodic memory decline and hippocampal atrophy than *BDNF* ^Val^ homozygotes, despite no differences in the rates of Aβ accumulation [8]. Further, these results are consistent with animal studies, which have shown that the secretion of mature BDNF is crucial in the neuronal integrity of the hippocampus [2], [20], and that Aβ decreases BDNF levels by reducing phosphorylated cAMP response element binding protein, which in turn regulates *BDNF* transcript expression [5]. Human and animal neuropathological studies have also found that interactions between *BDNF* Val66Met and Aβ-related synaptic changes occur in the hippocampus, and that these changes are related directly to memory [2], [20], [21]. Finally, genetic databases do not identify *BDNF* Val66Met polymorphism as increasing risk for AD [22]. Taken together, these data suggest that while *BDNF* Val66Met is unrelated to the presence of Aβ or to its accumulation, it may moderate the extent to which Aβ affects brain structure and memory function, at least in the prodromal stages of AD.

An important caveat of the current study is that the AIBL study is not an epidemiological sample. The selection of MCI groups was biased towards the inclusion of individuals with aMCI, and participants were predominantly highly educated, and had few existing or untreated medical or psychiatric illnesses. As such, it would be important for these findings to be replicated in adults with MCI and high Aβ in population-based studies, such as the Mayo Clinic Study of Aging, where it is possible that the effect of the *BDNF* Val66Met polymorphism on Aβ-related decline may be greater than that observed here. A second caveat is that we only investigated indirect interactions between *APOE*, *BDNF* Val66Met, Aβ and cognitive decline in adults with aMCI. This was primarily due to the small sample size, equivalence in the proportion of *APOE* ε4 carriers in *BDNF* ^Met^ carriers and *BDNF* ^Val^ homozygotes, and the previous observation that *APOE* ε4 does not interact with BDNF levels to affect cognitive function [23]. However, further investigation of the relationship between *APOE*, *BDNF* Val66Met, and Aβ on change in cognition needs to be conducted in larger, prospective studies of *APOE* allele groups [24], [25]. Finally, due to small sample sizes, we were unable to investigate whether *BDNF*Val66Met polymorphism has an effect on cognitive decline in individuals with MCI and low Aβ. However, as individuals with MCI and low Aβ do not show memory decline over 18 or 36 months [12], [14], and as we have previously shown that in healthy older adults, *BDNF*Val66Met polymorphism only exerts its effects on cognitive function and brain structure in individuals with high Aβ [8], we hypothesize that the *BDNF*Val66Met polymorphism would not have an effect on cognitive function and hippocampal volume in individuals with MCI and low Aβ.

Notwithstanding this limitation, results of the current study provide preliminary support for high Aβ and *BDNF* Val66Met polymorphism as important prognostic markers of increased memory decline and hippocampal atrophy in individuals with prodromal AD [13]. Further, as pharmacologically increasing BDNF levels in AD mouse models can ameliorate synaptic dysfunction and improve memory [26], and increasing BDNF secretion through aerobic exercise have been shown to improve memory performance in humans at risk for AD [27], interventions geared toward increasing BDNF levels may be a potential therapeutic strategy for the early stages of AD.
